# Manipulation of Life-History Decisions Using Leptin in a Wild Passerine

**DOI:** 10.1371/journal.pone.0034090

**Published:** 2012-03-20

**Authors:** Luc te Marvelde, Marcel E. Visser

**Affiliations:** Department of Animal Ecology, Netherlands Institute of Ecology (NIOO-KNAW), Wageningen, The Netherlands; University of Cordoba, Spain

## Abstract

Seasonal timing of reproduction and the number of clutches produced per season are two key avian life-history traits with major fitness consequences. Female condition may play an important role in these decisions. In mammals, body condition and leptin levels are correlated. In birds, the role of leptin remains unclear. We did two experiments where we implanted female great tits with a pellet releasing leptin evenly for 14 days, to manipulate their perceived body condition, or a placebo pellet. In the first experiment where females were implanted when feeding their first brood offspring we found, surprisingly, that placebo treated females were more likely to initiate a second brood compared to leptin treated females. Only one second brood fledged two chicks while five were deserted late in the incubation stage or when the first egg hatched. No difference was found in female or male return rate or in recruitment rate of fledglings of the first brood, possibly due to the desertion of the second broods. In our study population, where there is selection for early egg laying, earlier timing of reproduction might be hampered by food availability and thus nutritional state of the female before egg laying. We therefore implanted similar leptin pellets three weeks before the expected start of egg laying in an attempt to manipulate the laying dates of first clutches. However, leptin treated females did not initiate egg laying earlier compared to placebo treated females, suggesting that other variables than the perceived body condition play a major role in the timing of reproduction. Also, leptin treatment did not affect body mass, basal metabolic rate or feeding rates in captive females. Manipulating life history decisions using experimental protocols which do not alter individuals' energy balance are crucial in understanding the trade-off between costs and benefits of life history decisions.

## Introduction

Life-history theory predicts that key events in an organism's lifetime are organized in a way to yield maximum fitness [1]. Here we will focus on the number of breeding attempts within a season and timing of reproduction which are two of these key elements that are strongly shaped by natural selection.

### Multiple breeding attempts

Multiple breeding attempts within the same breeding season is a common reproductive tactic in a variety of taxonomic groups, including birds [2]. In great tits (*Parus major*), a facultative multiple breeding species, early breeding pairs are more likely to initiate a second clutch [3]. Second clutches are mostly initiated just before or just after fledging of the first brood. Because the fledged nestlings receive about three weeks of parental care outside the nest box [4], parental care has to be divided between the first and second brood once a second brood is initiated. Reduced parental care could lead to reduced survival of the first brood fledglings, but total recruitment could increase if fledglings of the second brood recruit in the population. Indeed, experimental removal of second broods in great tits resulted in increased breeding success of recruits of the first brood chicks the year after fledging [2]. Removal of second clutches also showed that female, but not male, survival increased, showing a cost to having multiple breeding attempts [5], and experimental brood enlargement of the first brood in blue tits (*Parus caeruleus*) resulted in a reduced probability of initiating a second brood [6].

It is unknown which cues are used in the decision to initiate a second brood. So far, one study was able to promote second brooding. Lõhmus and Björklund [7] showed, using a leptin manipulation in great tits, that increased perceived body condition at the end of the first brood increased the proportion of second broods. Unfortunately, no fitness effects were recorded.

### Timing of reproduction

Many bird species time their reproduction in a way that the period of maximum food requirements (feeding nestlings) coincides with the timing of maximum food availability (arthropod food peak). Breeding too late or too early has negative effects in terms of energy expenditure during chick feeding [8], [9] and fitness [10], [11]. In order to match the timing of food availability and food requirements, egg laying has to be initiated weeks before the occurrence of the food peak. Multiple environmental cues are used in the timing of reproduction. Besides photoperiod as initial predictive cue, supplementary cues like temperature are used to time reproduction [12]–[14]. In years with high spring temperatures, birds initiate timing of breeding earlier in the season. It could be that temperature in the period before egg laying itself is used as a cue. Alternatively, body condition before egg laying may be used as the cue [15]. For example, female mallards (*Anas platyrhynchos*) in good body condition breed earlier compared to females in poor body condition [16]. Since food availability and foraging success both increase as temperatures increase in spring [17], it is likely that an increase in body condition is confounded with the increase of temperature. Experimental manipulation of the body condition is needed to study the effects of body condition on timing of reproduction.

Studying the fitness costs of advanced reproduction is particularly interesting in the Hoge Veluwe study population of great tits, because climate change caused the seasonal timing of the phenology of the food peak to shift earlier in the season the last four decades. Great tits have advanced egg laying, but have not advanced adequately to match the shift of their food peak [10], [18]. We have two hypotheses for the lack of shift in timing of breeding. First, it is possible that great tits have not changed the way they use temperature as a cue (‘*cue hypothesis*’). Temperatures in early spring (the period when great tits have to decide to start laying eggs) have not increased as much as temperatures in late spring (the period which determines the timing of the caterpillar peak). Therefore, the correlation between temperatures in early and late spring have changed. If great tits still use the old rules to predict the timing of the caterpillar food peak using temperatures in early spring temperature, they will start egg laying too late. This hypothesis implies that egg laying could commence earlier, if the rules to predict timing of the food peak change. In this case, an advance of egg laying would lead to an increase in fitness due to the better match with the food peak. An alternative hypothesis as to why great tits lay their eggs too late is that early egg laying is constrained by food availability (‘*constraint hypothesis*’). Earlier egg laying would result in a higher workload, with likely negative effects of survival [19]. In this case, the fitness benefits of a better synchrony between the food peak and the nestlings' nutritional needs do not outweigh the decrease in fitness costs of earlier egg laying. Therefore, if the constraint hypothesis is true, advancing egg laying would lead to a decrease in fitness. Only experimental advancement of timing of reproduction could reveal potential fitness costs of increased energetic costs of early egg laying [20] and whether great tits in our study area are maladapted due to climate change [21]. Providing supplementary food could increase body condition and affect timing of reproduction [22], [23], but this is of limited use when the focus of the study is to measure fitness effects of early reproduction because supplementary food alters the energy balance of adults; birds do not have to work for their food and do not pay the costs of foraging [24]. Relaxing foraging effort by providing supplementary food before and during egg laying could potentially mask fitness costs of early egg laying on parental survival or fledgling recruitment when carry over effects of increased work load in the early stages of the reproductive cycle exist [19], [25]. Instead, to measure fitness effects, the perception of body condition should be altered without influencing the food availability. This could be done by experimentally elevating leptin levels (see below).

### Leptin

Most of our knowledge on the function of the hormone leptin comes from mammalian studies. In mammals, leptin is produced by fat cells and plays a major role in the energy balance and reproduction of mammals [26]. Leptin is involved in a negative feedback loop that regulates food intake and body weight [27]. Leptin levels increase after food is consumed [28], and thus serves as a short term energy balance signal. Leptin is also a long term signal of body condition as leptin levels circulating in the plasma correlate with body fat content [26]. In mammals, leptin binding to its receptor in the hypothalamus activates the sympathetic nervous system to increase energy expenditure [29]. Leptin also plays a major role in reproduction in mammals [26]. It is thought that leptin might have a role in signaling the nutritional status to the hypothalamus, which controls the reproductive neuroendocrine function [30].

The function of leptin has been studied in other vertebrates, including birds. Leptin in birds is a topic of debate after the initial cloning of leptin from chicken DNA could not be replicated [31], [32] and its existence questioned [33]. Pitel *et al.* [34] suggested the possibility that the leptin gene got lost through evolution while the leptin receptor still exists. Many studies with sometimes contrasting results have added to the discussion. Increased levels of leptin can cause a decrease in food intake as shown in both domesticated and wild bird species [35]–[37]. Immunisation against leptin in chicken resulted in increased food intake and increased daily weight and fat gain [38]. Leptin receptors have been found in ovaries and brain of laying hens [39]. Leptin advances puberty in chicken [40], can directly control basic chicken ovarian functions [41] and is involved in follicle maturation [26]. No leptin activity was found in Bar-tailed godwits (*Limosa lapponica*) and Adélie penguins (*Pygoscelis adeliae*) even though these two species undergo large changes in body fat in their annual cycle [42]. Kordonowy *et al.* [43] showed that leptin in free living starlings (*Sturnus vulgaris*) followed a seasonal pattern, with highest leptin levels during egg laying declining towards chick rearing. Unfortunately, leptin concentration in the weeks before egg laying were not included in this study. Increasing leptin levels in the period before egg laying might thus increase a bird's perceived body condition and might therefore affect the timing of reproduction in populations with selection for early reproduction.

### Aims and hypothesis

The aims of this study were threefold. First, we studied whether elevated leptin levels during the end of the first brood affects the decision to start a second brood. We assessed the fitness consequences of having a second brood in terms of parental survival, number of fledglings produced and fledgling recruitment. We expected females with experimentally elevated leptin levels to be more likely to produce second broods as in [7]. We expected survival of nestlings of first broods to be lower for those females producing second broods [2], because only the males will provide parental care after fledging. The extra workload (for males taking care of fledglings alone, for females the costs of producing eggs and incubation) could lead to reduced adult survival. Our second aim was to study whether elevated leptin in the period before egg laying affects the timing of egg laying and to assess whether there are fitness consequences of altered timing of reproduction. We expected early egg laying by leptin treated females as a result of the increased perceived body condition. Nestling recruitment would be higher for leptin treated females as they would then be timed better with the food peak, while adult survival is reduced because of the increased costs of early egg laying and incubation. The third aim of this study was to study the effect of elevated leptin levels on body mass, foraging activity and basal metabolic rate (BMR) in captive great tits, because an increase in energy expenditure or a decrease of food intake or body mass as a result of increasing leptin levels could potentially delay timing of breeding and thus affect the results for the previous aim. We expected leptin to decrease body mass as a consequence of decreased foraging activity (as has been shown in great tits) and to increase BMR (as has been found in mammals).

## Results

### Field experiment – Second broods

**Figure 1:**
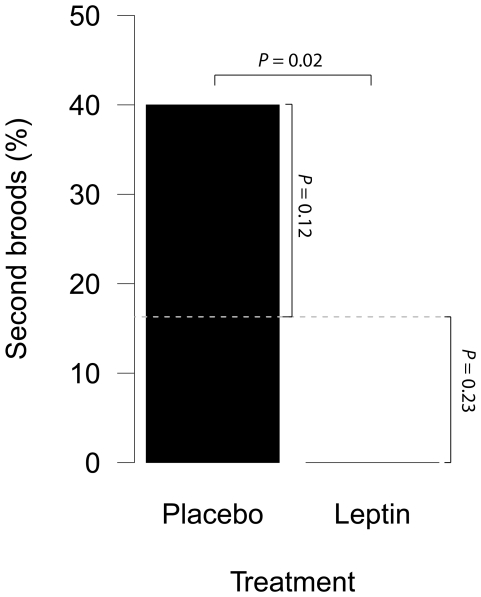
The effect of leptin on second brooding in wild great tits. Percentage of placebo and leptin treated females that initiated a second brood. The grey dashed line is the natural percentage of second broods in the year of the experiment for females with similar laying dates as the experimental females (7 out of 43, 16.3%). *n* = 15 for each treatment.

Against our expectation, placebo treated females were more likely to initiate a second breeding attempt compared to leptin treated females (placebo: 6/15 (40%), leptin: 0/15 (0%); Pearson's Chi-squared test with Yates' continuity correction; χ^2^_1_ = 5.21, *P* = 0.022; Fig. 1). The proportion of second broods from placebo treated females was more than double that of non-experimental females with similar laying dates (7 out of 43; 16.3%). However, this difference is not significant (Pearson's Chi-squared test with Yates' continuity correction; χ^2^_1_ = 2.36, P = 0.12). Leptin treated females were not less likely to initiate second broods compared to females with similar laying dates (Pearson's Chi-squared test with Yates' continuity correction; χ^2^_1_ = 1.45, *P* = 0.23).

Clutch sizes of second broods of placebo treated females were smaller compared to the clutch size of their first broods (paired t-test: t = 9.71, df = 5, *P* = 0.0002), but not different from other second broods of non-experimental great tits in 2009 (Two Sample Wilcoxon rank sum test W = 25.5, *P* = 0.47). Only one of the six second broods fledged (two) chicks. One nest was deserted early in the incubation stage and one after ∼10 days of incubation (of which the embryos were almost fully developed). The other three nests were deserted from the moment the first egg hatched. Seven out of 11 (64%) non-experimental females with a second brood were able to fledge nestlings.

Females which produced a second brood were likely to have invested more energy in reproduction compared to females which did not produce a second brood. These extra costs could result in reduced survival. However, eight leptin females and eight placebo females (of which three produced a second brood) were recorded in 2010 as breeding adults, thus showing no difference in survival to the next breeding season between leptin and placebo treated females. Within the experimental females, survival of females that initiated a second brood was not different from females that did not initiate a second brood (3/6 vs. 13/24.; Pearson's Chi-squared test with Yates' continuity correction χ^2^_1_ = 0.08, *P* = 0.78).

During incubation of the second clutch and possibly also during egg laying, females are not able to assist in caring for the first brood fledglings. Therefore, males may have worked harder to compensate for the female's absence, or alternatively compromise parental care at the costs of nestling survival. Five males of the control pairs and seven males of the leptin group (note that males in neither of the two groups were implanted a leptin pellet) returned as breeding males the next year (5/15 vs. 7/15; Pearson's Chi-squared test with Yates' continuity correction; χ^2^_1_ = 0.14, *P* = 0.71). Males of which females started a second brood were not less likely to survive than males of females which did not start a second brood (2/6 vs. 10/24; χ^2^_1_ = 0.009, *P* = 0.93).

A possible reduction in female provisioning behaviour as a result of initiating a second brood could have negative consequences for the fledglings of the first brood. However, six fledglings from the control group and six fledglings of the leptin group returned in 2010 as first year breeders out of a total of 297 fledglings (leptin *n* = 143 and placebo *n* = 154). None of the recruits came from second broods. First broods of females which initiated a second brood were not less likely to recruit as a breeder the next year compared to fledglings of females which did not initiate a second brood (2/59 vs. 10/238; Pearson's Chi-squared test with Yates' continuity correction; χ^2^_1_ = 0.007, *P* = 0.93).

### Field experiment – Timing of egg laying

**Figure 2:**
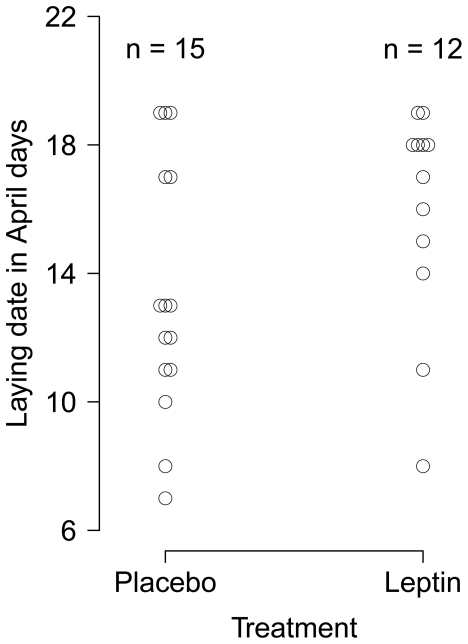
The effect of leptin on laying dates in wild great tits. Laying dates (date the first egg of the clutch is laid) of females implanted with a pellet releasing leptin for 14 days (2 µg leptin day^−1^ gram bodymass^−1^) or implanted a placebo pellet. Note that points were slightly separated to show overlapping data points.

Twelve out of 19 leptin females and 15 out of 18 placebo females laid eggs in the study area. One leptin treated female incubated an empty nest and thus was excluded from the analysis. Mean laying date of leptin treated females (*n* = 12, mean = 16.1 SD = 3.3) did not differ from mean laying dates of placebo treated females (*n* = 15, mean = 13.4, SD = 3.9; Two sample Wilcox rank sum test: W = 121.5, *P* = 0.13; Fig. 2). We were therefore not able to estimate the effect of a better match with the food peak on nestling recruitment and the effect of the likely increase in energetic costs during egg laying and incubation on adult survival.

### Aviary experiments – Body mass, feeding frequency and BMR

**Figure 3:**
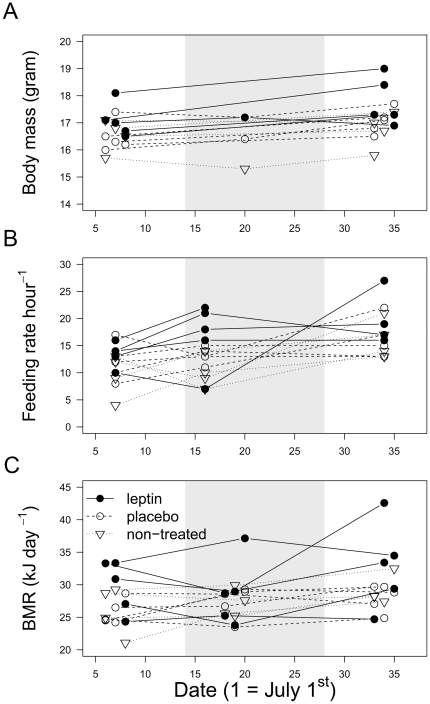
Effect of leptin on body mass, feeding frequency and BMR in captive great tits. Within-individual changes in body mass (grams; measured between 10AM and 10.30AM; panel A), number of visits to the feeding during the first hour after feeding (panel B), and basal metabolic rate (kJ day^−1^; panel C) for leptin, placebo and non-treated captive female great tits. The grey window represents the 14 day period during which leptin was released from the implanted pellet in the leptin group (2 µg leptin day^−1^ gram bodymass^−1^).

Linear mixed models with female as a random effect to control for multiple measures per female showed that leptin treatment did not affect within-female measurements of body mass (period * treatment interaction where period is before, during and after treatment; df = 4, F = 2.05, *P* = 0.12), feeding frequency of female great tits in the first hour after placing the food (period * treatment interaction; df = 4, F = 0.43, *P* = 0.78) or basal metabolic rate (BMR; period * treatment interaction; df = 4, F = 0.69, *P* = 0.61; Fig. 3).

## Discussion

We tested the effect of leptin on the number of broods within a season and timing of reproduction by implanting female great tits with pellets, which slowly released leptin for a period of 14 days, at different stages of the breeding cycle. Leptin treated females were less likely to initiate a second brood compared to placebo treated females. However, neither leptin nor placebo treated birds differed in their likeliness to initiate a second brood compared to non-experimental females of similar laying dates. We showed that leptin had no effect on the timing of reproduction. Also, we showed that leptin treatment did not affect body mass, feeding frequency or basal metabolic rate in captive female great tits.

In contrast to our expectations, placebo treated females were more likely to initiate second breeding attempts within the same season compared to leptin treated females. Our data are directly opposite to the data of Lõhmus and Björklund [7] who showed that a higher proportion of leptin treated females initiated second broods. In fact, our experiment was an exact replica of their experiment using the same species, with leptin from the same origin, in the same dosage and with pellets made by the same company. Although the function of leptin is mammals is well understood [26]–[30], its function in birds is still poorly understood and a long lasting topic of debate [31]–[34]. This study adds to the uncertainties of the role of leptin in birds.

The fact that five out of six second broods were deserted in the incubation stage or directly after hatching suggests that the placebo females were tricked into initiation of a second breeding attempt while they had not intended to do so, while 7 out of 11 (64%) non-experimental females with a second brood were able to fledge nestlings. Therefore we explored the possibility that the leptin and placebo pellets were accidentally swapped. However, in a bioassay, leptin activity was found in the residues of the bag in which the leptin pellets were stored (2.5 pg/ml) and no leptin activity was found in the bag in which the placebo pellets were stored (pers. comm. A. Gertler). Thus there is no evidence that the pellets were swapped. Another possible explanation, also explaining why placebo treated females had a higher rate of double brooding than non-experimental females, is that capturing and implanting females may affect their estimation of predation pressure and hence they may shift to more reproductive output, unless when they are in good condition (as perceived by the leptin implanted birds).

Although leptin treated females were less likely to produce second broods, this did not gain an advantage in terms of male and female return rate or increased recruitment of their first brood fledglings. This is in contrast to earlier experiments where Verhulst [5] showed that removing second broods lead to increased female, but not male, survival in years with low winter food availability. This could be due to our low sample size making it difficult to detect fitness effects if present. Alternatively, since all but one female deserted during or after incubation, females could have returned to help out after deserting, mitigating the negative fitness effects.

In 2010, a new batch of leptin and placebo pellets was used to test the effect of leptin on timing of reproduction. The window for timing of egg laying is set by photoperiod, which has a direct effect on gonadal development [13]. Besides photoperiod, secondary cues are used to fine tune timing of reproduction to annual variation in the optimal timing of reproduction [44]. Food availability in the period before egg laying is one of those secondary cues, as has been shown by supplementary feeding experiments [22]. There might be a direct effect of food availability on timing of reproduction, or an indirect effect via body condition. We found no effect of leptin on laying dates in great tits, which implies that perceived body condition in the weeks before egg laying does not play a major role in the timing of reproduction. Unfortunately, little is known about food availability in the period before egg laying. It is possible that earlier breeding is simply not possible energetically since early breeding means egg laying under colder conditions with increased energetic costs [20]. If the costs of early egg laying are higher than the benefits of a better match with the food peak, being mismatched can be the optimal strategy [21].

It is possible that leptin caused an increase in energetic costs in great tits, as a lack of leptin in mammals results in decreased energy expenditure [45]. If leptin increased energy expenditure in great tits, this could potentially explain why none of the leptin treated females started a second brood or why we found no effect on laying dates. However, measurements of BMR of captive female great tits showed no evidence of an effect of leptin. To our knowledge, this is the first study measuring the effect of leptin on BMR in a wild bird species. Human studies on leptin suggest that leptin affects energy balance mainly through the regulation of food intake and not via a direct effect on energy expenditure [46]. In our study, leptin did not affect feeding rates although depressed food intake after leptin injection has been found in mammals [47], in domesticated chicken [48] and wild great tits [36], albeit higher concentrations of leptin were used compared to our study. Also, Lõhmus *et al.* [36] showed a decrease in feeding rates in the first 40 minutes after injection, after which it became equal to the control group. Slow release of leptin at a dosage of 2 µg per gram body mass per 24 h did not affect feeding rates in our study. Consequently, it also did not affect body weight and we therefore think the lack of second breeding attempts within the same season and the lack of effect of leptin on timing of reproduction were not caused by increased costs as a result of the leptin treatment itself.

Life history decisions often involve a trade-off between current reproductive investment and future reproductive investment. Therefore, research should focus on manipulations which do not affect the energetic costs of current reproduction (e.g. supplementary feeding) as this disrupts the balance between costs and benefits in current reproduction investments. Manipulations of timing of reproduction without changing the costs associated with early reproduction are particularly important in understanding the consequences of climate change, since climate change has already, and will continue, to advance the seasonal timing of arthropod food sources for forest living insectivorous animals. Without understanding the ultimate fitness costs of early reproduction, predicting evolutionary responses to climate change are difficult to make.

## Methods

### Ethics Statement

The experiments reported here comply with the current law in The Netherlands and were carried out under licenses of the Animal Ethics Committee of the Royal Netherlands Academy of Arts and Sciences (KNAW; leptin specific licenses: CTE 09.05 & NIOO 10.01; general field work license: NIOO 10.07).

### Study area and study species

This study consists of two field experiments (2009, 2010) and one experiment with captive female great tits (2010). The field experiments were carried out in the National park ‘De Hoge Veluwe’ (52°02′07″N 5°51′32″E). The study area consists of 171 ha of mixed woodland on poor sandy soils, dominated by oak and pine with about 400 nest boxes. Each year, up to 130 boxes are occupied by great tits, a small (∼18 grams) passerine bird species.

### Standard field protocol

Nest boxes were checked weekly from the beginning of April to monitor nest building, egg laying and incubation. Nests were visited daily from two days before the expected hatching date to ensure the exact hatching date (date at which at least one chick hatched ( = day 0)). When the nestlings were seven days of age, parents were caught using a spring loaded trap inside the nest box while feeding the nestlings. Parents were ringed with a uniquely numbered aluminium ring as well as with three colour rings coding a unique colour combination. Also, all nestlings were ringed with a uniquely numbered aluminum ring. Nest boxes were checked after fledging to record possible dead nestlings and unhatched eggs.

### Field experiment – Second broods

During the breeding season in 2009, 30 female great tits were caught while feeding nestlings (of 10 days old) using a spring trap inside the nest box (10 females per day on three consecutive days). Each day, five females were assigned to the placebo treatment and five to the leptin treatment. The leptin/placebo was administered in a custom made pellet (Innovative Research of America, Sarasota, Florida, USA; 3 mm in diameter; 16 mg) which continuously released chicken recombinant leptin (provided by A. Gertler, Protein Laboratories Rehovot Ltd., Rehovot, Israel) over a 14 day period. The leptin pellets were made of a matrix (mainly cholesterol) and 500 µg recombinant chicken leptin. As the matrix slowly dissolves leptin is released (about 2 µg per gram body mass per day). The placebo pellets were made of only the matrix and contained no leptin. Pellets (of the 2009 batch) were inserted subcutaneously in the field in the adult female when the nestlings of her first brood were 10 days old (fledging normally occurs at the age of 18 or 19 days).

After the implantation event the original box and surrounding nest boxes were checked twice times a week to monitor the initiation of second broods. Incubating females were identified based on their colour combination to ensure it had started a second brood. From then onwards, the protocol was the same as for the first broods.

### Field experiment – Timing of egg laying

On March 22^nd^ and 23^rd^ 2010, 37 females great tits were implanted with a leptin or placebo pellet (leptin: *n* = 19; placebo: *n* = 18; using the 2010 batch of pellets). Their egg laying date was monitored via the standard field protocol.

### Aviary experiment – Body mass, foraging activity & basal metabolic rate (BMR)

In 2010, 15 captive female great tits originating from the Hoge Veluwe population were used to study the effect of leptin on body mass, foraging activity and BMR. These females were hand reared in 2009 from 10 days old. Since independence these females were housed indoors in controlled conditions. Females were already ringed with one uniquely numbered aluminum ring and were given a unique combination of two colour rings to make identification on the video possible to score foraging behaviour (see below). From the end of May, all females were housed at the previous location of the Netherlands Institute of Ecology (Heteren, The Netherlands; 51°57′20″N–5°44′34″E) in one large outdoor aviary under natural light and temperature conditions and *ad libitum* food and water. Females were randomly assigned to the leptin or placebo treatment or not to be treated (*n* = 5 for each group). Leptin and placebo pellets (from the 2010 batch) were implanted on June 14^th^.

We measured basal metabolic rate (BMR) in terms of oxygen consumption in an open-circuit respirometer. BMR was measured during three periods: before, during and after the treatment (July 7/6/8, July 18/19/20 and August 2/3/4 respectively; five females per night randomly spread over the treatments). Females were weighed to the nearest 0.1 g using an electronic balance (Sartorius PT 1500, USA) before going into the respirometer chamber. Birds were isolated in five sealed respirometer chambers (0.76 l) and placed in the darkness of a climate cabinet (Sanyo MIR-553, Sanyo E&E Europe BV, Etten-Leur, The Netherlands) at 25°C (i.e. within their thermoneutral zone), always between 11PM and 1130PM. H_2_O and CO_2_ were removed from the inlet air (blown into the respirometer chamber) respectively with Drierite® (6 mesh, Sigma-Aldrich Chemie BV, Zwijndrecht, The Netherlands) and Soda lime ® (Sigma-Aldrich Chemie BV, Zwijndrecht, The Netherlands). Air flow rate was set to 250 ml min^−1^ with flowmeters (Brooks Instrument B.V., Ede, The Netherlands) previously calibrated using a soap bubble method (Bubble-O-Meter, LLC, Dublin, OH, USA). Oxygen content of outlet air was measured every 30 seconds with an oxygen analyser (Servomex 4100, Servomex BV, Zoetemeer, The Netherlands). Oxygen consumption (ml O_2_ min^−1^) was calculated as the difference in oxygen concentration between air from the respirometer chambers and reference air from an empty chamber. As only one oxygen analyzer was used, measurements alternated between the five experimental and one reference chamber every 15 minutes. Measurements over the last 5 minutes of each 15 minute period were averaged and the period with the lowest averaged oxygen consumption was used to calculate BMR. The oxygen consumption was converted to metabolic rate (kJ 24 h^−1^) by assuming an energetic equivalence of 20 kJ L^−1^ O_2_.

Feeding rates were scored on the same captive female great tits by analyzing video recordings made of the feeding table before, during and after the leptin treatment (respectively July 11^th^, 16^th^ and August 3^rd^). On each of the mornings, around 8.30AM, all left-over food of the previous day was removed. Fresh food was presented in a single tray and filmed with a digital video camera (JVC Everio, Germany). Females were identified based on their colour rings. Feeding frequencies for individual females were scored during the first hour after presenting the food. Only visits were food was taken were used in the analyses (541 of 579 visits (93.4%)).

### Statistics

Statistical tests are named in the results. All statistics were done using R version 2.10.1 [49].
